# The ecological validity of traditional standing and novel bicycle balance and agility tests for predicting performance in mountain bikers

**DOI:** 10.1016/j.smhs.2022.10.003

**Published:** 2022-10-09

**Authors:** K. Buchholtz, M. Lambert, T.L. Burgess

**Affiliations:** a Health, Physical Activity, Lifestyle, and Sport (HPALS) Research Centre, University of Cape Town, Cape Town, South Africa; b Department of Physiotherapy, LUNEX International University of Health, Exercise and Sports, Luxembourg; c Division of Physiotherapy, University of Cape Town, Anzio Road, Observatory, Cape Town, South Africa; d Centre for Medical Ethics and Law, Faculty of Medicine and Health Sciences, Stellenbosch University, Cape Town, South Africa

**Keywords:** Bicycling, Control, Proprioception

## Abstract

Falls are a common mechanism of injury in mountain biking and may be related to a loss of control of the bicycle. Traditionally, the components of bicycle control (balance and agility) are measured in standing and running, which may not reflect the skills required in mountain biking. In this paper, we present the validity of both traditional standing and novel bicycle-specific balance tests in mountain bikers. Twenty-nine male and female participants completed indoor laboratory tests and an outdoor downhill trail. Participants completed single-leg stance balance, Y-balance test, one static and four dynamic bicycle-specific balance tests, a bicycle agility test, and an outdoor downhill trail. Single-leg stance balance and Y-balance tests with eyes open had poor validity when associated with bicycle control. The static (*r* ​= ​−0.57, *p=* 0.001) and four dynamic bicycle balance tests (*r* ​= ​−0.51 to −0.78, *p=* 0.005 to 0.0001), and the bicycle agility test (*r* ​= ​0.87, *p<* 0.0001) had moderate to strong relationships with the outdoor downhill run. Single-leg stance balance and Y-balance tests with eyes open are not valid measures of performance on a mountain bike, and should not be used to assess these populations. Our novel bicycle balance tests have adequate validity to be used as measures of performance in mountain bikers.

## Abbreviations

Ant: Anterior
cm: centimetre
*CI*: Confidence Interval
CS: Composite Score
CV_TEM_: Standardised Coefficient of Variation
DBBT: Dynamic Bicycle Balance Test
HREC REF: Human Research Ethics Committee Reference Number
IAT: Illinois Agility Test
*ICC*: Intraclass Correlation Coefficient
kg: kilogram
km⋅h^−1^: kilometres per hour
m: metre
NCS: Normalised Composite Score
NS: Normalised Score
PL: Posterolateral
PM: Posteromedial
s: seconds
*SD*: Standard Deviation

## Introduction

Falling off a bicycle is the most common mechanism of sustaining a sudden onset injury in mountain biking and usually occurs following the loss of control of the bicycle.^1^ Bicycle control may be influenced by many factors, including balance, agility, reaction time and fatigue.^2^^,^^3^ Good bicycle control (the ability to maintain balance, speed and directional control of the bicycle) may contribute to both increased performance of the cyclist and the reduced risk of falling and subsequent injury.^2^ Traditionally, balance and agility clinical assessments have been performed with the participant in a standing position or while running around objects.^4^, ^5^, ^6^ Typically, athletes have been assessed using single leg stance for static balance, and the Y-Balance test (or Star Excursion Balance Test) for dynamic balance. These tests are reliable and valid for standing and running-related activities but whether they can assess balance and agility in mountain bikers is not known. Therefore, we developed static and dynamic bicycle balance tests, and a bicycle-specific agility test, for use by clinicians working with mountain bikers. The validity of these tests has not been established.

The property of validity defines whether a test measures what it claims to measure.^7^ Ecological validity is the relationship between a phenomenon in its natural setting and the investigation of this phenomenon in an experimental context, for example, in a laboratory.^8^ In the context of control of a mountain bike, if a test determines bicycle control to be good in a laboratory setting, and this is correlated to the performance of the cyclist in natural mountain biking setting, this test would have good ecological validity.^9^

The aim of this study was to determine the ecological validity of traditional standing balance tests and novel bicycle balance tests in the assessment of mountain bikers. We hypothesised that traditional standing balance tests, specifically, single-leg stance balance and Y-balance tests with eyes open, would not be valid measures of bicycle control in mountain bikers.

## Materials and methods

This study design was a descriptive, correlational study to assess the ecological validity of traditional and novel balance and bicycle-specific agility tests.

Male and female cyclists aged 18–60 years were recruited through advertisements on social media sites (Facebook, Twitter, and Instagram) related to cycling, cycling clubs, cycling coaches and sports physiotherapy practices in the local region for this study. A prerequisite for recruitment was that participants were accustomed to cycling on mountain bikes with clipless pedals and cleats. This was used as a proxy for sufficient experience to safely complete the indoor testing component of the study. No minimum weekly training hours or kilometres were specified, with the intention of recruiting participants with diverse cycling ability. Any cyclists with an injury or pain at the time of the study were excluded from the study. Participants with known balance deficiencies or neurological disorders (as previously diagnosed by a medical practitioner) that may affect balance, and those with vision impairments were excluded to avoid potential confounding factors.

The study was approved by the Human Research Ethics Committee of the Faculty of Health Sciences, University of Cape Town (HREC REF: 268/2018). All participants provided written informed consent before participation.

Participants attended three indoor testing sessions (between Monday and Friday), and an outdoor session on the weekend of the same week. They completed the American Heart Association/American College of Sports Medicine pre-participation screening tool to assess for medical fitness,^10^ and a previously validated demographic and training history questionnaire before testing commenced.^11^^,^^12^ Anthropometric measurements (including mass, stature, and skin folds) and vision screening were performed. During the indoor testing sessions, participants completed traditional standing balance tests for static and dynamic balance: the single leg stance test and the Y-Balance test. The single leg stance test was performed with the eyes open, and time (s) was measured until control was lost, indicated by the participant placing the other foot on the ground. The test was terminated at 45 ​s (based on the normative values determined by Springer et al.^4^) if the participant had maintained control for the full period. Participants were tested on each leg.^4^ The Y-Balance test was measured as maximal reach in anterior, posterior-medial and posterior-lateral directions on each leg and normalised to the participant's leg length. The Y-Balance test is reported as a normalised score (NS) relative to the length of the leg (as a percentage), and as normalised composite score (NCS) as a combined score for all three directions of movement for each leg. The reliability of these traditional balance tests has been previously established.^4^^,^^6^^,^^13^ Participants then completed novel bicycle balance and agility tests.

### Static bicycle balance test

**Fig. 1:**
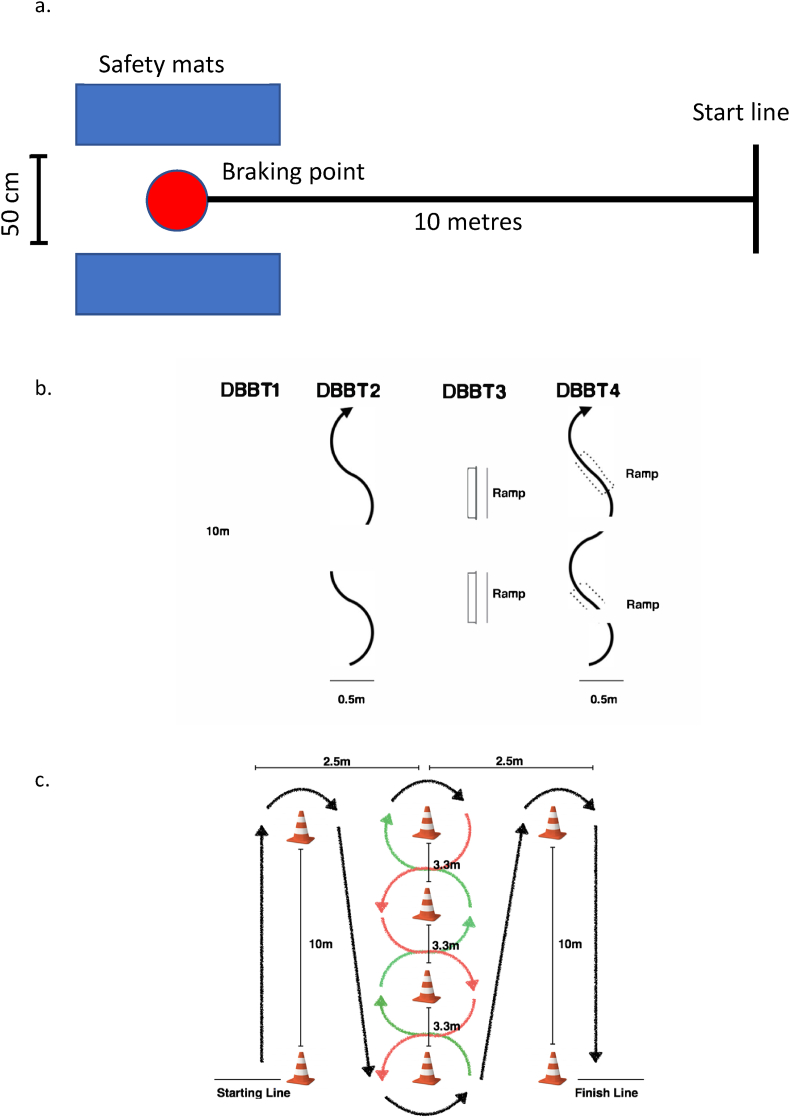
Layout of novel bicycle balance and agility tests: 1a) Static bicycle balance test; 1b) Dynamic bicycle balance tests; 1c) Bicycle specific agility test. Adapted from: Raya et al., 2013. (Legend: cm ​= ​centimetres, DBBT ​= ​dynamic bicycle balance test, m ​= ​metres).

The static bicycle balance test is a novel test we designed to assess balance in a functional cycling position. The participants performed the test from a moving start position behind the start line on their own bicycle to allow them to clip their feet in before increasing speed (Fig. 1a). They rode in a straight line for 10 ​m from the start line accelerating maximally to increase speed over the 10 ​m distance, before rapidly braking between two safety mats (measuring 30 ​cm high, and a marked point on the ground (braking point). The ground contact point of the front wheel had to line up with the marking on the ground. They braked sharply and had to maintain their position on the bike for the maximal period possible. The test was terminated if the cyclist removed one or both hands from the handlebars, one or both feet from the pedals, or turned the wheel more than 10 ​cm to either side of the neutral position (to prevent track standing). Reliability of the test has previously been established (*CV*_TEM_ ​= ​28.1; *ICC* ​= ​0.59).^11^

### Dynamic bicycle balance tests

The dynamic bicycle balance tests (DBBT) are a battery of four novel tests performed at low speeds to assess the ability of the participant to control the bicycle whilst moving (Fig. 1b). The DBBTs progressed from simple to complex over four increasingly difficult tests. In DBBT1 and DBBT2, the cyclist followed a straight line and curved line respectively. In DBBT3 and DBBT4, they followed these same lines over two ramps (30 ​cm wide, 100 ​cm long and 20 ​cm high at the apex/highest point) positioned on the lines. All tests were performed at slow speed; the participants were asked to move as slowly as possible and attempt to maintain the speed at least between 5 and 7 ​km⋅h^−1^ for all four tests, and even slower if they were able to. This speed was based on the minimum speed possible for inexperienced cyclists to maintain balance, as described by Cain et al.,^14^ and translates into a time of 5.2–7.2 ​s to ride the 10 ​m line. A Brower Timing System (Brower Timing Systems, Draper, UT, USA) was used to record the time taken to complete each test (in seconds and milliseconds). The timing system was aligned with marked ‘start’ and ‘end’ lines on the floor and these were identified on the video analysis to synchronise the accuracy and timing measurement.

A GoPro Hero sports camera was mounted under the front handlebar overlooking the front wheel, to record the participant's ability to keep the wheel on the line. The camera had a wide-angle lens and was able to view the wheel and the line on the floor clearly. The percentage of time spent on the line (accuracy score) was calculated using the video analysis after the completion of the tests. Reliability of these tests has been previously established (*ICC* ​= ​0.65–0.81; *effect size* ​= ​0.00–0.15 [no effect to trivial effect]).^11^ The DBBT's are reported as a composite score of time and accuracy, and the best performance by each participant (over the three testing days) was used for analysis.^11^ To calculate the score, time and accuracy were converted to z-scores by standardising to a mean of zero and a standard deviation of one. These arbitrary units were then averaged to generate a composite score (*CS*) of the time and accuracy measures. The CS was used for analysis.

### Bicycle-specific agility test

The bicycle specific agility test was adapted from the Illinois Agility Test (IAT) designed for running.^5^ The layout of the course is shown in Fig. 1c. The participants moved around poles of 1.5 ​m height marking the course. They were instructed to move as quickly as possible around the poles. Time taken to complete the task (in seconds) was recorded using a Brower Timing System (Brower Timing Systems, Draper, UT, USA). The test was terminated if the participant put a foot on the ground, touched one of the poles or moved incorrectly around the course. This test has previously established excellent reliability (*CV*_TEM_ ​= ​3.4; *ICC* ​= ​0.95; *effect size* ​= ​−0.20 to 0.05 (trivial to small)).^11^

### Procedure

Participants were familiarised on each test at each testing session to reduce the impact of a ‘learning effect’.^15^^,^^16^ Three familiarisation attempts were given each day and verbal feedback on their speed and performance was provided. Following the familiarisation, each test was performed three times on each day of testing, and the best performance on each day was used for analysis.

### Downhill technical trail

The outdoor testing session consisted of three attempts at a downhill technical trail in the Newlands forest, Cape Town. This occurred on the final testing day during the week. All participants had attempted the trail at least three times before the testing day to ensure competence and safety, and to reduce a possible learning effect on the trail. The downhill trail was 440 ​m in length from top to bottom, with an elevation change of 38 ​m. The average gradient was 8.6% with the steepest on the downhill being 39.6%. The ground surface was hard-packed soil, with areas of rock garden and narrow pathways to pass through. The time (s) to complete the section was recorded for each trial and the fastest time of the three successful attempts was used for analysis. Timing was performed using a stopwatch, with one researcher at each end of the trail communicating via two-way radios to start the timing as the participants began. The outdoor testing was only done during periods of non-extreme weather, and weather conditions were recorded on the day of testing to monitor consistency of the conditions (mean ​± ​*SD* of average daily temperature: 15.5° ​± ​3.3°; maximum daily temperature: 22.1° ​± ​5.3°; average wind speed: 16.3 ​± ​6.4 ​km⋅h^−1^; and humidity 66.8% ​± ​13.1%).

Participants used their own bicycle, and shoes for testing and no changes to the bicycle configuration (including tyre and suspension pressures) during the testing period were made. Participants used their own bicycles to ensure their maximal comfort and optimised positioning, and to prevent differing configurations of a new bicycle interfering with their performance.

### Statistical analysis

Descriptive analytics were performed. A Shapiro-Wilk test was performed to assess normality, and data were found to be normally distributed. In the absence of a true ‘gold standard’ test, the outdoor test was defined as the test which assessed real-world performance and a measure of ecological validity.^9^ A Pearson's correlation was calculated to assess whether the outdoor tests correlated with the results from the previous tests, thereby assessing their ecological validity.^17^

## Results

Thirty-two participants were recruited for the study, but two participants withdrew due to illness and transport related issues, and one participant was lost to follow up (final *n* ​= ​29; seven females; mean ​± ​*SD*; age ​= ​34.9 ​± ​10.9 years; body mass ​= ​75.2 ​± ​9.1 ​kg; stature ​= ​176.1 ​± ​7.6 ​cm; body fat ​= ​21.1% ​± ​7.0%). Nineteen participants rode mountain bicycles with full suspension, and ten rode with front suspension only. Twenty-six of the bicycles were 73.7 ​cm (29-inch) wheel size, and the remaining were 69.9 ​cm (27.5-inch).

All participants trained regularly on a mountain bike, with 20 participants also cycling on a road bike. They reported an average of 9.5 and 14.0 years of mountain, and road cycling experience, respectively. They cycled a median of four times a week. We analysed the data from the male and female participants as one group because the relationship in performance between the tests was unlikely to be affected by sex.

In the single leg stance test, all participants were able to maintain the 45 ​s required for the maximum time in the described test, for both right and left legs. No correlations were therefore able to be calculated for the single leg stance test. The results of all the physical tests are shown in Table 1. The best overall performance for each physical test was used to calculate the correlation.

**Table 1:** Best performance for each variable and correlations between physical tests and the outdoor skills test.

Variable	Best performance	*r*-value (95%*CI*)	*p*-value
**Y-balance R Ant NS (%)**	87.5 ​± ​7.5 (72.9–100.6)	0.06 (−0.31-0.42)	0.74
**Y-balance L Ant NS (%)**	87.5 ​± ​7.2 (74.4–99.1)	0.02 (−0.35-0.39)	0.90
**Y-balance R PM NS (%)**	119.1 ​± ​14.4 (87.9–152.9)	−0.07 (−0.43-0.30)	0.70
**Y-balance L PM NS (%)**	117.4 ​± ​13.8 (93.8–154.8)	0.00 (−0.36-0.37)	0.99
**Y-balance R PL NS (%)**	111.71 ​± ​16.9 (70.2–152.4)	−0.07 (−0.43-0.30)	0.71
**Y-balance L PL NS (%)**	114 ​± ​17.4 (85.6–159.1)	−0.01 (−0.37-0.36)	0.98
**Y-balance R NCS (%)**	92.4 ​± ​14.1 (61.7–132.2)	−0.26 (−0.57-0.12)	0.18
**Y-balance L NCS (%)**	94.5 ​± ​14.6 (65.3–140.1)	−0.16 (−0.50-0.22)	0.39
**Static bike balance test (s)**	2.36 ​± ​0.90 (0.10–4.02)	−0.57 (−0.77–−0.25)	**0.001*∗***
**DBBT 1 CS**	0.22 ​± ​0.57 (−1.15-1.49)	−0.51(-0.74- 0.17)	**0.005*∗***
**DBBT 2 CS**	0.25 ​± ​0.62 (−1.54-1.75)	−0.56 (−0.81–−0.34)	**0.0003*∗∗***
**DBBT 3 CS**	0.30 ​± ​0.54 (−0.77-1.56)	−0.52 (−0.75–−0.20)	**0.0035*∗***
**DBBT 4 CS**	0.3 ​± ​0.67 (−1.13-1.86)	−0.78 (−0.89–−0.54)	***<*0.0001*∗∗***
**Bicycle agility (s)**	28.4 ​± ​3.8 (22.5–38.4)	0.87 (0.73–0.94)	***<*0.0001*∗∗***
**Outdoor technical run (s)**	88 ​± ​24 (54–169)	–	***-***

Legend: R ​= ​right, L ​= ​left, Ant ​= ​Anterior, PM=Posteromedial, PL=Posterolateral, NS=Normalised Score, NCS=Normalised Composite Score, CS=Composite Score, DBBT ​= ​Dynamic Bicycle Balance Test, *CI*=Confidence Interval, s ​= ​seconds***∗ p* ​< ​0.05*, ∗∗p* ​< ​0.001**.

**Fig. 2:**
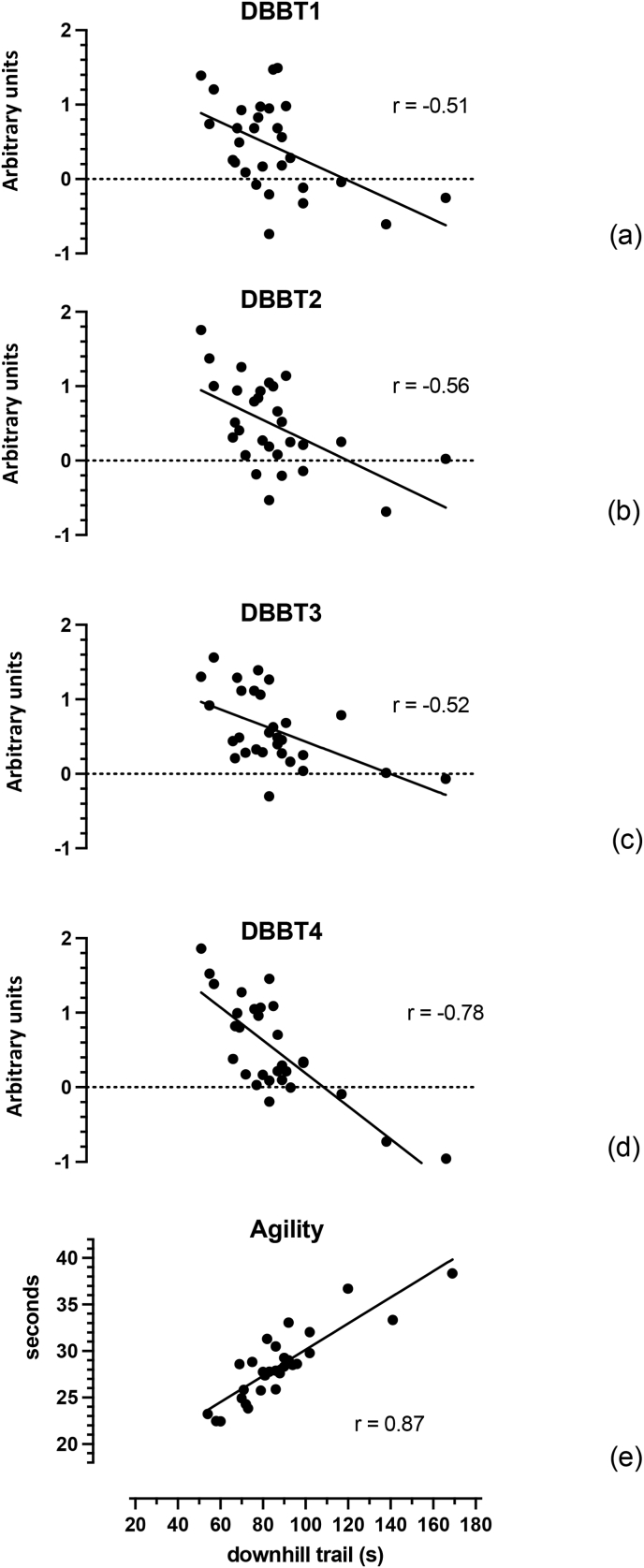
Correlations between Dynamic Bicycle Balance Tests (DBBT) DBBT1 (a), DBBT2 (b), DBBT3 (c), DBBT4 (d) and the bicycle agility test (d), and the downhill trail. (Legend: s ​= ​seconds).

There were no significant associations between the Y-balance tests and the outdoor downhill trail (Table 1). The static bike balance test and DBBTs 1 to 3 had significant moderate negative correlations (Fig. 2). The DBBT4 and bicycle agility test had strong negative and positive associations to the outdoor test, respectively (Fig. 2).

## Discussion

Clinicians use physical tests to assess balance and agility in athletes as measures of both performance and potential risk of injury.^4^, ^5^, ^6^ These are traditionally performed in standing and running and have not previously been assessed for their validity in cycling populations. Few other studies have assessed balance in cyclists, and balance on a bicycle.^2^^,^^14^^,^^18^^,^^19^ These studies have been performed in technologically advanced laboratories making it challenging to translate the findings for clinicians and coaches in an exercise and sports medicine environment. None of the previous studies specifically measured balance on a mountain bike and have instead focussed almost exclusively on commuter cycling.^2^^,^^14^^,^^19^ In our study we assessed the ecological validity of two traditional balance tests (single leg stance and Y-Balance test) and novel bicycle balance and agility tests against actual performance riding a mountain bike on a downhill trail. We confirmed our hypothesis that these traditional standing balance tests were not valid measures of bicycle control in mountain bikers. Our novel tests performed well against the outdoor trail, in particular, the DDBT4 and bicycle-specific agility tests.

Traditional standing balance tests had poor relationships to cycling performance. Neither the single leg stance test nor the Y-balance tests were able to provide information on cycling performance in a mountain biking population. These tests are therefore not appropriate measures of balance and agility in mountain bikers and should not be used for these purposes.

The static bicycle balance test had a significant moderate relationship with the outdoor test but has previously displayed greater variability between testing days.^11^ This lack of reliability affects the use of these tests in this population.

All four dynamic bicycle balance tests had significant relationships to the performance on the outdoor technical run. The DBBT4 had the strongest relationship of the balance tests indicating that as the composite score on the DBBT4 improved, the time taken to descend the outdoor technical run decreased (i.e., performance improved).

These DBBTs are reliable and valid and can be reproduced at low cost. A prerequisite for the test is having a sufficiently large space (greater than 10 ​m ​× ​5 ​m) with a constant surface for conducting the tests.

The bicycle agility test had a strong positive relationship with the outdoor downhill run indicating a strong association to outdoor performance on a bicycle. As agility performance improved (shorter time), the time taken to descend the outdoor trail decreased. All measures of reliability^11^ and validity support that this test has value as a indicator of outdoor mountain biking performance.

To our knowledge, no previous studies have assessed bicycle-specific agility. Agility is a complex psychomotor skill, incorporating acceleration, deceleration, change of direction and decision making.^20^ These skills are used while navigating a bicycle through the changing and varying terrain in mountain biking. The assessment of agility may provide information on the cyclist's ability to control their bicycle, and in our study, we found agility to be strongly related to outdoor mountain biking performance. Our modification of the Illinois Agility test^5^ is easily administered, and considering all our tests, has the strongest relationship with the outdoor performance. The test can be performed in any space large enough for the bicycle to move around the marker poles. Further long-term prospective research should be performed to determine whether performance on the bicycle-specific agility test, or other factors (for example physical and cognitive fatigue) are related to risk of falling or acute injury.

Clinical tests need to be both reliable (or repeatable) and valid, which would indicate that the tests are assessing what they purport to assess.^21^ In the development of novel tests, it is challenging to assess validity when there is no gold standard test as a source of comparison.

While the novel tests are valid and reliable measures of performance on a mountain bike, we did not establish the relationship between these tests and risk of injury. Prospective studies investigating the relationship between bicycle-specific balance and agility, and the risk of injury need to be conducted.

### Limitations and recommendations

We specifically recruited a heterogenous population of cyclists to improve the external validity of the findings. This allowed for an overall assessment of validity of the tests, but not the assessment of the validity in specific ages, sexes or sub-categories of cycling. Further research needs to investigate specific groups to confirm whether the relationships identified in this study remain once stratified into these categories. These analyses would require larger sample sizes in future.

Cyclists were able to ride their own bicycles, with their own personal settings for saddle height, tyre pressure, wheel size, suspension pressures and suspension type. This was to prevent the impact of using an unfamiliar bicycle for balance and agility tests on their performance, and the analyses assessed the paired performances of the participants against each test in the test battery. These bicycle-related components may have resulted in some confounding of the results. Further research is needed to identify the amplitude of the impact from these potential confounders.

A logistical challenge is that the video analysis of the DBBTs is arduous and time-consuming (approximately two to 3 ​min per video), requiring analysis on a frame-by-frame basis of the videos.^11^ Automating this video analysis would improve the likelihood of the test being used by clinicians. The low cost and easily reproducible nature of the tests make them more accessible for use in a clinical setting than previous studies using force plates and 3D video analysis laboratories.^2^^,^^14^^,^^18^ The timing of the outdoor trail was performed using two researchers communicating via radio feedback. An automated method of timing could improve the accuracy of this measure.

## Conclusion

Traditional standing balance tests, specifically, single-leg stance balance and Y-balance tests with eyes open, are not valid measures of performance on a mountain bike, and should not be used to assess these populations. Our novel bicycle balance tests have adequate measures of reliability and validity to be used as measures of performance in mountain bikers. Further research should be conducted to determine the use of these tests as assessments of risk of injury.

## Practical implications

• Traditional standing balance tests (single-leg stance balance and Y-balance tests with eyes open) are not associated with the performance and control on a bicycle and are not recommended as an assessment for a mountain biking population
• The novel dynamic bicycle balance tests and the bicycle-specific agility tests are valid indicators of bicycle control and can be used in a clinical mountain biking population.
• The novel tests are accessible and easy to administer tests of mountain biking performance and can be recommended for use in assessing bicycle control.

## Submission statement

We confirm that this manuscript has not been published elsewhere and is not currently under consideration by another journal. All authors have approved the final manuscript and agree with its submission to this journal.

## Ethical approval statement

The study was approved by the Human Research Ethics Committee of the Faculty of Health Sciences, University of Cape Town (HREC REF: 268/2018). All participants provided written informed consent before participation.

## Authors' contribution

KB conceived of the study, collected and analysed the data, and wrote the manuscript. ML and TB conceived of the study and provided supervision of the study. ML analysed the data together with KB. All authors read and approved the final manuscript.

## Conflict of interest

The authors declare that they have no known competing financial interests or personal relationships that could have appeared to influence the work reported in this paper.
